# Clinical and CT Radiomics Nomogram for Preoperative Differentiation of Pulmonary Adenocarcinoma From Tuberculoma in Solitary Solid Nodule

**DOI:** 10.3389/fonc.2021.701598

**Published:** 2021-10-12

**Authors:** Yaoyao Zhuo, Yi Zhan, Zhiyong Zhang, Fei Shan, Jie Shen, Daoming Wang, Mingfeng Yu

**Affiliations:** ^1^ Department of Radiology, Shanghai Public Health Clinical Center, Fudan University, Shanghai, China; ^2^ Department of Radiology, Zhongshan Hospital, Fudan University, Shanghai, China; ^3^ Research Institute of Big Data, Fudan University, Shanghai, China; ^4^ Fudan University, Shanghai, China; ^5^ Department of Research and Development, Shanghai United Imaging Intelligence Co., Ltd., Shanghai, China; ^6^ Department of Thoracic Surgery, Beilun Second People’s Hospital, Zhejiang, China

**Keywords:** lung adenocarcinoma, tuberculoma, radiomics, CT features, nomogram

## Abstract

**Aim:**

To investigate clinical and computed tomography (CT) radiomics nomogram for preoperative differentiation of lung adenocarcinoma (LAC) from lung tuberculoma (LTB) in patients with pulmonary solitary solid nodule (PSSN).

**Materials and Methods:**

A total of 313 patients were recruited in this retrospective study, including 96 pathologically confirmed LAC and 217 clinically confirmed LTB. Patients were assigned at random to training set (n = 220) and validation set (n = 93) according to 7:3 ratio. A total of 2,589 radiomics features were extracted from each three-dimensional (3D) lung nodule on thin-slice CT images and radiomics signatures were built using the least absolute shrinkage and selection operator (LASSO) logistic regression. The predictive nomogram was established based on radiomics and clinical features. Decision curve analysis was performed with training and validation sets to assess the clinical usefulness of the prediction model.

**Results:**

A total of six clinical features were selected as independent predictors, including spiculated sign, vacuole, minimum diameter of nodule, mediastinal lymphadenectasis, sex, and age. The radiomics nomogram of lung nodules, consisting of 15 selected radiomics parameters and six clinical features showed good prediction in the training set [area under the curve (AUC), 1.00; 95% confidence interval (CI), 0.99–1.00] and validation set (AUC, 0.99; 95% CI, 0.98–1.00). The nomogram model that combined radiomics and clinical features was better than both single models (p < 0.05). Decision curve analysis showed that radiomics features were beneficial to clinical settings.

**Conclusion:**

The radiomics nomogram, derived from unenhanced thin-slice chest CT images, showed favorable prediction efficacy for differentiating LAC from LTB in patients with PSSN.

## Introduction

A pulmonary solitary solid nodule (PSSN) refers to an isolated round opacity with a well-defined margin and less than 30 mm in maximum diameter on computed tomography (CT) images (1). PSSN is more likely to be benign, either by routine screening or by accident (2, 3), but it may also be malignant at different stages. The prevalence of malignant solitary pulmonary nodule was documented to be 1.1%–12%, and lung adenocarcinoma (LAC) predominated (4, 5). However, lung tuberculoma (LTB) can also show malignant CT characteristics on CT, such as spiculated sign and pleural indentation, which is difficult to distinguish from LAC (6, 7). According to Lung Imaging Reporting and Data System (Lung-RADS) version 1.1, pulmonary solid nodule needs chest CT follow-up for 3–12 months, and further examination or puncture biopsy is suggested if the nodule is highly suspicious to be malignant (8). However, this standard recommendation will increase additional radiation injury and psychological and financial burden and may even miss the best treatment time. Therefore, a fast and effective method is needed to differentiate between LAC and LTB in PSSN.

Radiomics can describe the characteristics of the lesion by high-throughput extraction of a large number of image features, which is an emerging process with potential to promote better clinical decision-making (9, 10). The radiomics models have been proven to have good diagnostic efficacy in clinical applications of lung nodules, including differentiating between benign and malignant nodule, preoperative prediction of nodule type, or prognostic analysis (11–13). Imaging examination is one of the routine procedures of daily clinical diagnosis, so radiomics research is accessible. In addition, radiomics research has both temporal and spatial heterogeneity that not only can provide macroscopic images and local microenvironment of the lesion but also can reflect the progress of the lesion (14, 15). Two studies have focused on the differential diagnosis of LAC and LTB using U-net-based deep learning nomogram models (16, 17). However, the reproducibility and stability of CT radiomics features need further study and verification, which is affected by scanning parameters, reconstruction algorithms, and even region-of-interest (ROI) extraction methods (18–20).

In this study, we aimed to extract the radiomics parameters of PSSN and establish predictive nomogram models combined with clinical information to noninvasively identify LAC and LTB.

## Materials and Methods

### Patients

The Ethic Review Boards of Shanghai Public Health Clinical Center and Zhongshan Hospital have approved this retrospective study and waived the written informed consent. A total of 313 patients were recruited in this retrospective study from January 1, 2018, to March 30, 2020, at Shanghai Public Health Clinical Center and Zhongshan Hospital, including 96 pathologically confirmed LAC and 217 clinically confirmed LTB. The inclusion criteria of patients with LAC are as follows: (a) surgical pathology-confirmed adenocarcinoma; (b) unenhanced thin-slice (<2 mm) CT examination was performed within 2 weeks before surgery; (c) the maximum diameter of the nodule was less than 3 cm; (d) solitary solid nodule without calcification. The inclusion criteria of patients with LTB are as follows: (a) *Mycobacterium tuberculosis* was confirmed by culturing or assay from at least one respiratory specimen, including sputum, bronchoalveolar lavage fluid, and nasopharyngeal aspirate; (b) thin-slice (<2 mm) CT examination was performed; (c) the maximum diameter of the nodule was less than 3 cm; (d) solitary solid nodule without calcification. Patient who met any one of the following criteria was excluded in this study: (a) multiple pulmonary nodules; (b) LAC underwent neoadjuvant chemotherapy before surgery; (c) poor CT image quality, including artifacts or no continuous thin-slice images.

### CT Image Acquisition and CT Annotation

A total of two CT scanners were used to perform chest CT unenhanced examination: Somaton Force (SIEMENS, Germany) and Aquilion One/320 (TOSHIBA, Japan). The patient was in the supine position with both arms raised to reduce scanning artifacts from the shoulders and upper limbs. The locational marker was the sternoclavicular joint, and the scanning range was from the tip of the lung to the costophrenic angle. The CT scanning parameters were as follows: tube voltage, 120 kV; tube current, auto mA; pitch, TOSHIBA 0.813/SIEMENS 1; detector width, TOSHIBA 80 mm × 0.5 mm/SIEMENS 64 mm × 0.625 mm; rotation time, TOSHIBA 0.5 s/SIEMENS 0.75 s; matrix, 512 × 512; lung window settings (width/level), 1,200/-600 Hounsfield units (HU); and mediastinal window settings (width/level), 350/40 HU. The TOSHIBA CT images with 1-mm thickness and 0.5-mm (0.625 mm in SIEMENS CT images) interval were reconstructed using the lung algorithm.

**Figure 1:**
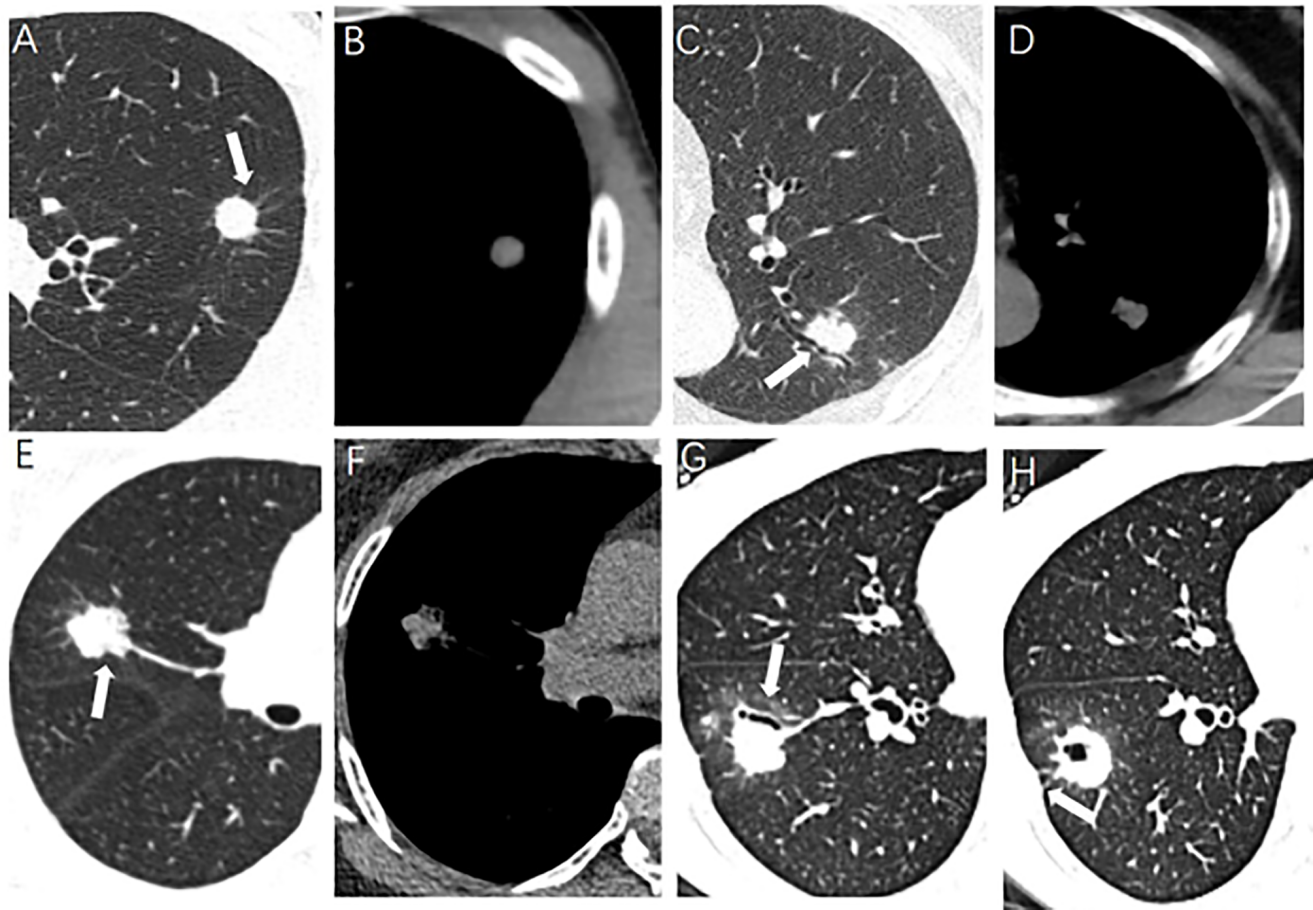
Some CT morphological characteristics of pulmonary solitary solid nodule. **(A, B)** A 30-year-old male, lung tuberculoma (LTB) with spiculated sign (arrow). **(C, D)** A 65-year-old female, LTB with air bronchogram (arrow). **(E, F)** A 62-year-old female, lung adenocarcinoma (LAC) with lobulated sign (arrow). **(G, H)** A 33-year-old female, LAC with air bronchogram and pleural indentation (arrow).

The morphological characteristics of PSSN on CT included the following: (a) size (maximum, minimum, and mean diameter); (b) spiculated sign, radially nonbranched linear shadows around the edge of the nodule (21); (c) lobulated sign; (d) boundary (clear or unclear); (e) cavity, a gaseous density with maximum diameter more than 5 mm; (f) vacuole, a gaseous density with maximum diameter less than 5 mm; (g) air bronchogram, the tubular gas-density bronchus reaches the edge of the nodule, entering or not entering the nodule; (h) pleural indentation, the pleura is pulled to form a triangular structure filled with fluid and connected to the lung lesion by a linear structure; (i) pulmonary vascular abnormalities, including vessel convergence and expansion; (j) mediastinal lymphadenectasis, short diameter more than 1 cm without calcification. Some CT morphological characteristics of PSSN were shown in **Figure 1**. Two experienced chest radiologists viewed the PSSN CT characteristics independently and were both blinded to the clinical data and pathological diagnosis. When there was disagreement, above two radiologists reached a consensus through discussion.

### Radiomics Feature Selection and Radiomics Signature Construction

**Figure 2:**
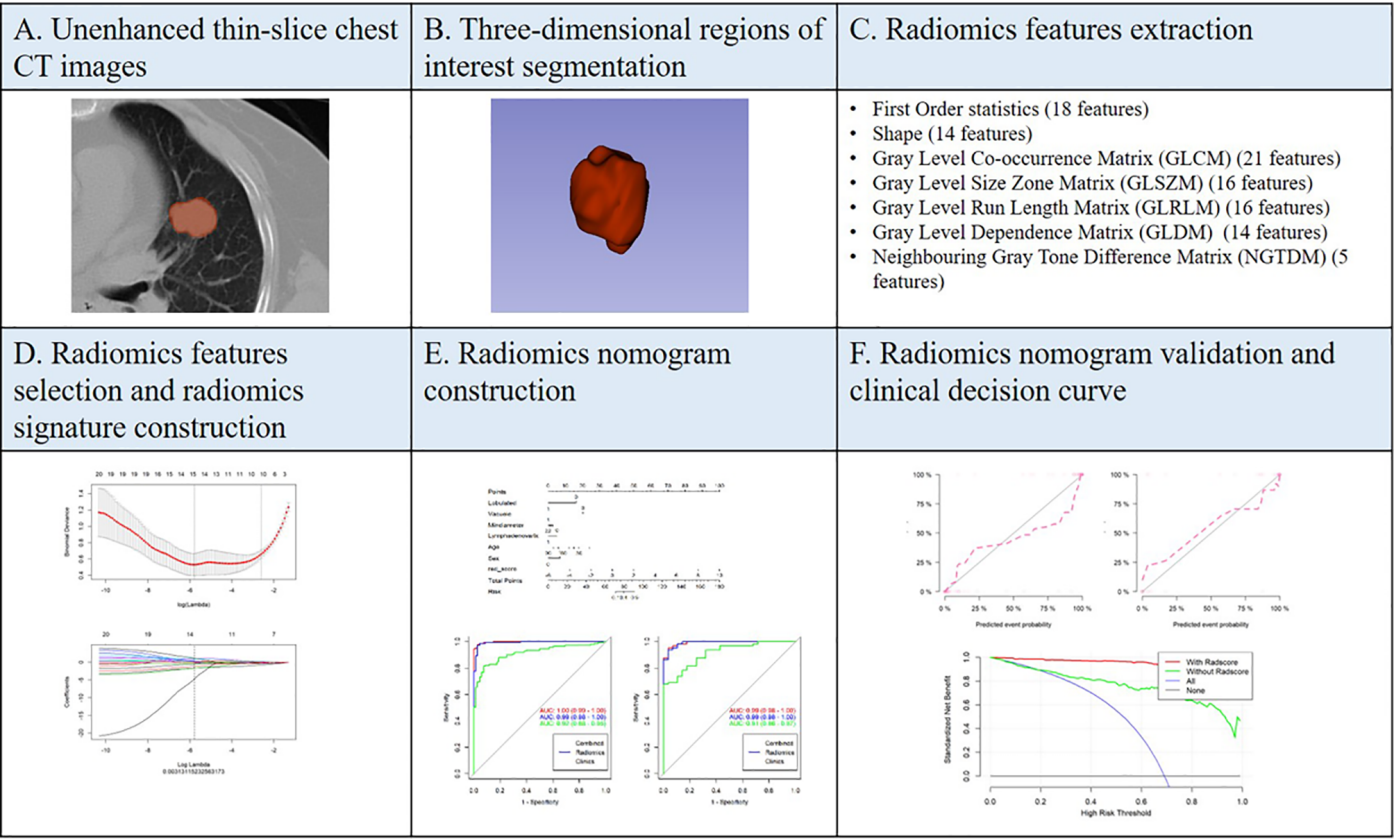
Overview of the study methodology.

Automatic PSSN segmentation was performed on a software called uAI Research Portal developed by Shanghai United Imaging Intelligence Inc. (http://urp.united-imaging.com:8080). Multiple deep learning models were used to identify chest structures and then automatically outline pulmonary nodules based on CT values and morphology (**Figure 2**). All automatically segmented three-dimensional (3D) regions of interest (ROIs) were reviewed by a radiologist and manually adjusted if necessary.

Radiomics feature extraction was performed on the uAI Research Portal. Radiomics features were divided into the following classes: (a) first-order statistics; (b) shape-based features; (c) high-order features, including gray-level co-occurrence matrix (GLCM), gray-level size zone matrix (GLSZM), gray-level run length matrix (GLRLM), gray-level dependence matrix (GLDM), and neighboring gray tone difference matrix (NGTDM). A total of 14 filters were used during the process of feature extraction, including boxmean, additivegaussiannoise, binomialblurimage, curvatureflow, boxsigmaimage, normalize, laplaciansharpening, discretegaussian, mean, specklenoise, recursivegaussian, shotnoise, laplacian of gaussian, and wavelet. Finally, a total of 2,589 radiomics features were extracted for each ROI.

Two feature selection methods, maximum relevance and minimum redundancy (mRMR) and least absolute shrinkage and selection operator (LASSO), were used to select radiomics features. At first, mRMR was performed to eliminate the redundant and irrelevant features; 30 features were retained after this step. Then, *LASSO* was conducted to choose the optimized subset of features to construct the final model. The number of the selected features was determined based on the regular parameter lambda, the most predictive subsets of features were chosen, and the corresponding coefficients were evaluated. Radscore was calculated by summing the selected features weighted by their coefficients. Patients were assigned at random to training set (n = 220) and validation set (n = 93) according to 7:3 ratio. We compared the radscores from LAC and LTB on training and validation sets, respectively. The receiver operating characteristic (ROC) curve was used to evaluate the performance of radiomics signature model.

### Radiomics Nomogram Construction and Validation

The clinical data were analyzed by univariate logistic regression analysis and multivariate logistic regression analysis to select the independent predictors of distinguishing between LAC and LTB. The clinical variables and selected radiomics features were combined to establish the radiomics nomogram. Clinical data and radiomics features were used separately to establish ROC curves both in training and validation sets, and the areas under the curves (AUCs) were calculated to evaluate the diagnostic efficacy. Finally, the calibration curves were built to evaluate the calibration ability of the nomogram both in training and validation sets.

To evaluate the clinical usefulness of radiomics features, a clinical decision curve was constructed using standardized net benefit and high risk threshold (22, 23).

### Statistical Analysis

The LASSO method constructed a penalty function by adding constraint conditions, and a prediction model was constructed by performing 10-fold cross validation. DeLong’s test was used between different ROCs, and the Hosmer–Lemeshow test was used to evaluate the goodness of fit of the nomograms.

For continuous variables (including age, nodule size), the Wilcoxon rank-sum test was used between two groups. The categorical variables (including sex, CT morphological characteristics) were compared with *x* ^2^ test. The ROC curve was used to evaluate clinical usefulness and calculate the cutoff values. The SPSS software (version 20) was used to perform all statistical analyses. The bilateral p-value <0.05 was considered statistically significant. Measurement data were expressed as mean ± standard deviation (SD).

## Results

### Clinical Characteristics

The clinical and CT characteristics were shown in **Table 1**. In this retrospective study, a total of 313 patients were recruited from two hospitals, including 96 pathologically confirmed LAC (44 males and 52 females; age 59.43 ± 11.49 years) and 217 clinically confirmed LTB (161 males and 56 females; age 45.49 ± 18.93 years). There were statistically significant differences in gender and age between the two groups: the age of LTB group was smaller than that of LAC group (p < 0.001), and the proportion of male patients was higher than that of LAC group (p < 0.001).

**Table 1:** The clinical and CT image characteristics of participants.

	All patients (n = 313)	Adenocarcinoma (n = 96)	Tuberculoma (n = 217)	p-value
Age (years)	49.76 ± 18.16	59.43 ± 11.49	45.49 ± 18.93	<0.001
Gender				<0.001
Female	108 (34.50)*	52 (54.17)	56 (25.81)	
Male	205 (65.50)	44 (45.83)	161 (74.19)	
Maximum diameter of nodule (mm)	15.66 ± 5.27	18.05 ± 6.06	14.61 ± 4.50	<0.001
Minimum diameter of nodule (mm)	11.92 ± 4.11	13.95 ± 4.82	11.01 ± 3.40	<0.001
Mean diameter of nodule (mm)	13.79 ± 4.50	15.99 ± 5.24	12.81 ± 3.75	<0.001
Spiculated sign				<0.001
Present	227 (72.52)	83 (86.46)	144 (66.36)	
Absent	86 (27.48)	13 (13.54)	73 (33.64)	
Cavity				0.255
Present	32 (10.22)	7 (7.29)	25 (11.52)	
Absent	281 (89.78)	89 (92.71)	192 (88.48)	
Vacuole				0.015
Present	20 (6.39)	11 (11.46)	9 (4.15)	
Absent	293 (93.61)	85 (88.54)	208 (95.85)	
Boundary				<0.001
Clear	306 (97.76)	89 (92.71)	217 (100.00)	
Unclear	7 (2.24)	7 (7.29)	0 (0.00)	
Lobulated sign				<0.001
Present	196 (62.62)	95 (98.96)	101 (46.54)	
Absent	117 (37.38)	1 (1.04)	116 (53.46)	
Air bronchogram				0.031
Present	177 (62.26)	63 (51.43)	114 (52.53)	
Absent	136 (37.74)	33 (48.57)	103 (47.47)	
Pleural indentation				0.287
Present	234 (74.76)	68 (70.83)	166 (76.50)	
Absent	79 (25.24)	28 (29.17)	51 (23.50)	
Pulmonary vascular abnormalities				0.065
Present	270 (86.26)	88 (91.77)	182 (83.87)	
Absent	43 (13.74)	8 (8.33)	35 (16.13)	
Mediastinal lymphadenectasis				<0.001
Present	105 (33.55)	16 (16.67)	89 (41.01)	
Absent	208 (66.45)	80 (83.33)	128 (58.99)	

*Data are numbers of patients, with percentages in parentheses.

For CT characteristics of 3D PSSN, the maximum, minimum, and mean diameter of LTB were all less than LAC (all p < 0.001). Compared with LTB, LAC was more prone to be with spiculated sign, lobulated sign, and vacuole (p < 0.001, p < 0.001, p = 0.015, respectively). On the other hand, LTB tended to have air bronchogram and mediastinal lymphadenectasis compared to LAC (p = 0.031, p < 0.001, respectively). In addition, the nodule boundaries of more LTB patients were unclear (p < 0.001). Some CT findings showed no statistically significant difference between the two groups, including cavity, pleural indentation, and pulmonary vascular abnormalities (p = 0.255, p = 0.287, and p = 0.065, respectively).

### Radiomics Feature Selection and Radiomics Signature Construction

**Figure 3:**
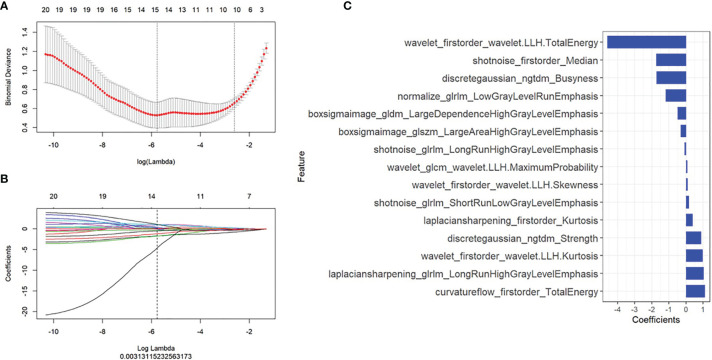
Radiomics features selection. The least absolute shrinkage and selection operator included **(A)** choosing the regular parameter λ and **(B)** determining the number of the feature. A total of 15 radiomics features were chosen **(C)**.

There were 2,589 radiomics features extracted for each ROI, and a total of 15 radiomics features with non-zero coefficients were selected based on the best lambda value and LASSO. The first-order statistics and high-order features with different filters were calculated as meaningful radiomics features, and the details of these selected features were shown in **Figure 3**. Radscore was calculated by summing the 15 selected radiomics features weighted by their coefficients and then a constant 1.469 was added (details in **Supplementary Material S1**).

We compared the radscore from LAC to LTB on training and validation sets, respectively, and ROC analysis was used to evaluate the performance of the radiomics signature model. The results showed that the radscores of patients with LAC were lower than those of patients with LTB, and they were statistically significant (p < 0.0000, p = 0.016, respectively) (**Supplementary Materials 2A, B**). ROC analysis showed good performance in the training set [AUC, 0.99; 95% confidence interval (CI), 0.98–1.00] and validation set (AUC, 0.99; 95% CI, 0.98–1.00) (**Supplementary Materials 2C, D**).

### Radiomics Nomogram Construction and Validation

According to the univariate and multivariate logistic regression analysis, six clinical parameters were selected as independent predictors, including sex, age, lobulated sign, vacuole, minimum diameter of nodule, and mediastinal lymphadenectasis (**Supplementary Materials 3, 4**).

**Figure 4:**
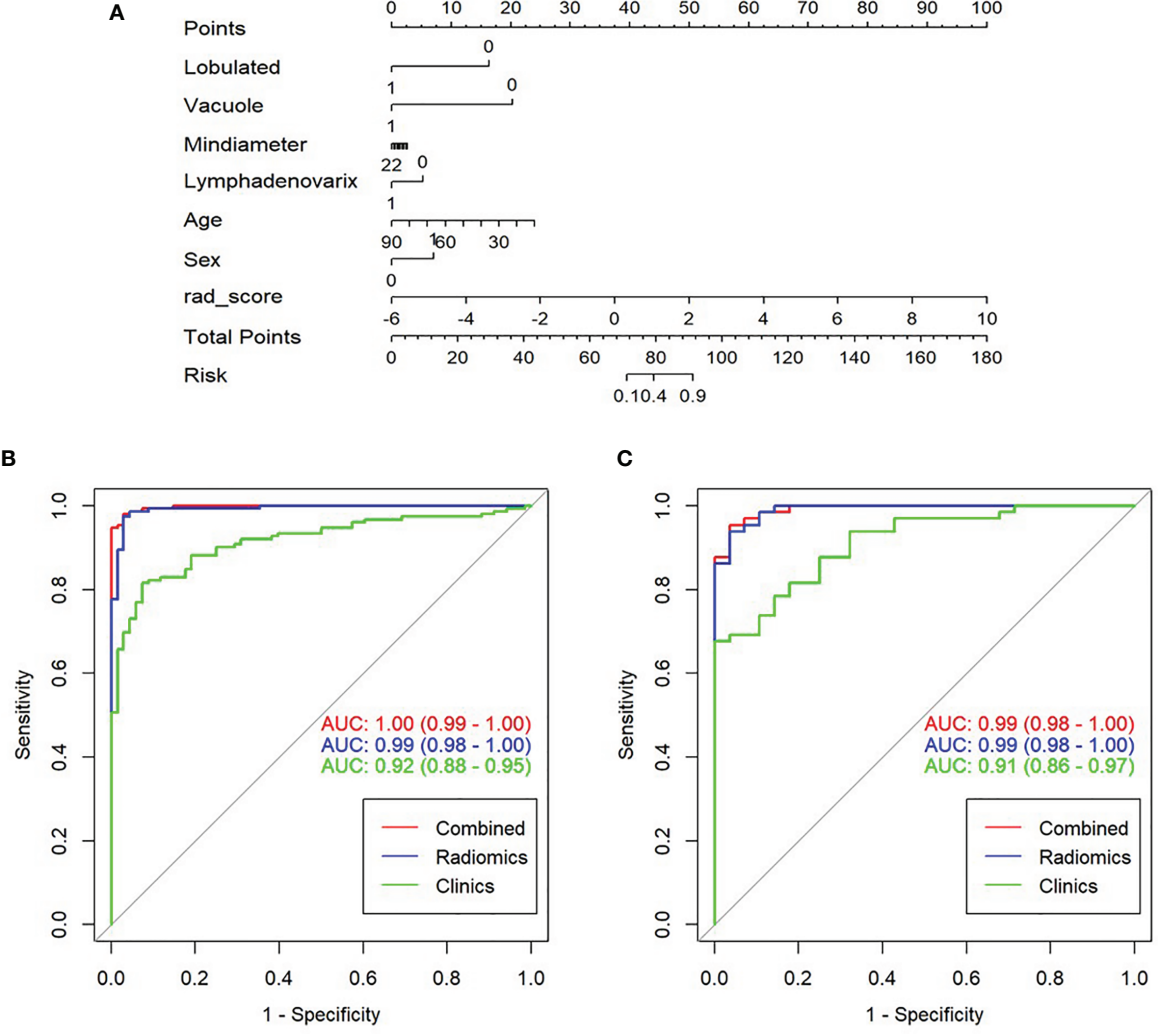
Construction, performance, and validation of radiomics nomogram. **(A)** The radiomics nomogram was developed using seven selected radiomics parameters and two clinical features. Receiver operating characteristic (ROC) curves of nomogram and clinical model in training set **(B)** and validation set **(C)**.

A nomogram model was built to distinguish between LAC and LTB based on multiple logistics regression equations (**Figure 4A**). ROC and decision curves were used to evaluate the clinical usefulness of the prediction model in both the training and validation sets. The radiomics nomogram of PSSN showed good prediction in the training set (AUC, 1.00; 95% CI, 0.99–1.00) and validation set (AUC, 0.99; 95% CI, 0.98–1.00) (**Figures 4B, C** **)**. In addition, the results also showed that the AUC of nomogram model were larger than those of clinical model and radiomics signature model in both training and validation sets, and there were statistically significant differences among the above three models in both training and validation sets (p < 0.001, p = 0.003, respectively). In other words, the nomogram model that combined radiomics and clinical features was better than both single models. The accuracy, sensitivity, and specificity of nomogram model in validation set were 0.957, 0.988, and 0.900, respectively (**Table 2**).

**Table 2:** The clinical model, radiomics signature, and radiomics nomogram for patients with LAC and LTB in training and validation sets.

Group	Model	AUC curve (95% CI)	Accuracy	Sensitivity	Specificity	PPV	NPV
Training set	Clinical model	0.92 (0.88–0.95)	0.8500	0.9612	0.6923	0.8158	0.9265
	Radiomics signature	0.99 (0.98–1.00)	0.9727	0.9706	0.9737	0.9429	0.9867
	Radiomics nomogram	1.00 (0.98–1.00)	0.9772	0.9868	0.9565	0.9803	0.9706
Validation set	Clinical model	0.91 (0.86–0.97)	0.7849	0.9245	0.6000	0.7538	0.8571
	Radiomics signature	0.99 (0.98–1.00)	0.9462	0.9643	0.9385	0.8710	0.9839
	Radiomics nomogram	0.99 (0.98–1.00)	0.9570	0.9841	0.9000	0.9538	0.9643

AUC curve, the area under receiver operating characteristic curve; CI, confidence interval; LAC, lung adenocarcinoma; LTB, lung tuberculoma; PPV, positive predictive value; NPV, negative predictive value.

**Figure 5:**
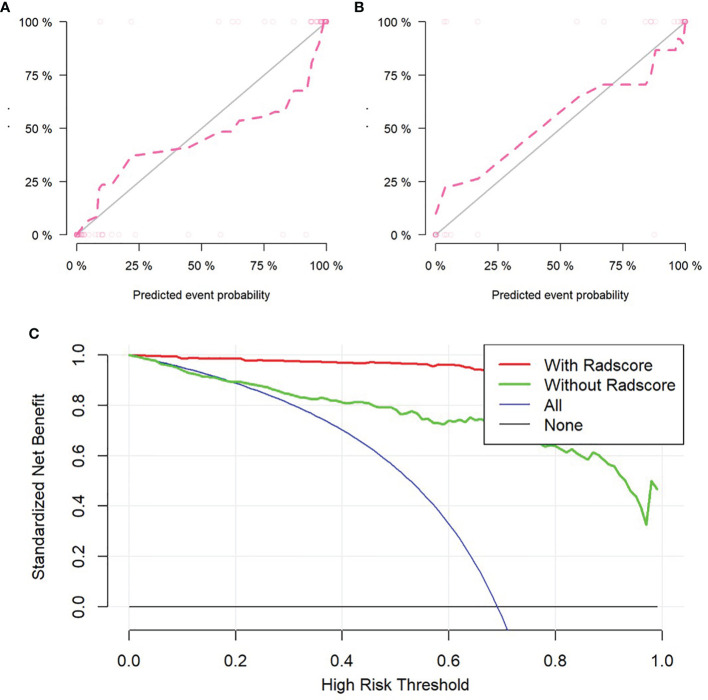
Validation of radiomics nomogram, the calibration curves of radiomics nomogram in training set **(A)** and validation set **(B)**. Decision curve analysis for radiomics and clinical model to evaluate the clinical usefulness of the model **(C)**.

The self-service method was used to resample the calibration curve 1,000 times to ensure the accuracy of the results. The result of Hosmer–Lemeshow test showed that p-value was 0.586 in training set, indicating that the model had good calibration ability. Similarly, the p-value was 0.074 in validation set, which also showed that the fitting degree of the model was good (**Figures 5A, B** **)**.

### Clinical Decision Curve

To evaluate the clinical usefulness of radiomics features, a clinical decision curve was constructed using standardized net benefit and high-risk threshold (**Figure 5C**). The clinical decision curve showed that both models with and without radscore could bring net benefits to patients compared to the case of treat-all and treat-none, of which the model with radscore added more benefits.

## Discussion

In this retrospective study, there were some significant differences in qualitative and quantitative clinical data between LAC and LTB patients with PSSN. A total of six clinical variables were selected to build the prediction nomogram model for differential diagnoses of LAC from LTB, including sex, age, lobulated sign, vacuole, minimum diameter of nodule, and mediastinal lymphadenectasis. The radiomics nomogram of PSSN, consisting of 15 selected radiomics parameters and above six clinical features, showed good predictive ability in both the training and validation sets. In addition, there were statistically significant differences among clinical model, radiomics signatures, and radiomics nomogram models. The nomogram model that combined radiomics and clinical features was better than both single models.

Some clinical and CT imaging features were related to the differential diagnosis of LAC and LTB in our study. The sex, age, lobulated sign, vacuole, minimum diameter of nodule, and mediastinal lymphadenectasis were selected to be independent predictors of PSSN. This study showed that the proportion of male patients with LTB was higher than that of LAC patients, which may relate to the different habits of smoking and tobacco use between males and females (24, 25). Patients with LAC had a higher age of onset than tuberculosis patients, which could be caused by decreased immunity, cell damage caused by long-term chronic disease, or an increased risk of genetic errors in the old. Malignant nodule was more likely to have a lobulated sign because of invasive growth of malignant cells in the pulmonary interstitium, which was consistent with previous research (26, 27). Vacuole sign refers to the presence of a small air-like low-density shadow within the nodule with smooth edges and no more than 5 mm. Vacuole sign is often seen in malignant nodules, which is an important sign of early lung cancer, but also occasionally seen in benign nodules (28, 29). There were statistically significant differences in nodule size between the two groups, and Chu et al. (30) found that larger pulmonary nodules (diameter >1 cm) had more malignant CT features compared with smaller nodules (diameter <1 cm) in patients with solid lung cancerous nodules. Patients with LTB tended to have mediastinal lymphadenectasis compared to LAC patients in our study, but Zhu et al. (31) found that malignant diseases were mostly in the diseases with mediastinal lymphadenectasis, and the benign diseases were mainly granuloma in a cohort study with 846 patients who underwent endobronchial ultrasound-guided transbronchial needle aspiration. In mediastinal lymphadenectasis caused by LAC or LTB for different reasons, tuberculosis patients were mostly due to lymph node tuberculosis, while lung cancer patients are often caused by cancer cell metastasis.

Univariate logistic regression analysis showed that spiculated sign, air bronchogram, and nodule boundary were statistically significant differences between the two groups, whereas these differences were not apparent on multivariate logistic regression analysis. The formation of spiculated sign is related to interlobular septal thickening, the lymphatic channels filled with malignant cells, or the fibrosis caused by the obstruction of peripheral blood vessels. Some studies indicated that spiculated sign was associated with lung cancer and could achieve moderate diagnostic performance (AUC = 0.76) for differentiating between benign and malignant lung nodules (16, 32). A meta-analysis revealed that CT-based spiculated sign alone was not sufficient to distinguish benign from malignant pulmonary nodules in clinical settings (32).

The AUCs of clinical model in training and validation sets were 0.88 and 0.86, respectively. The radiomics signature model and nomogram model of lung nodules showed better prediction in the training set and validation set, especially the nomogram model. A total of 15 non-zero coefficients were selected from lung nodules, including first-order statistics and high-order features (NGTDM, GLRLM, GLSZM, GLCM, and GLDM) with different filters. First-order statistics, also known as gray histogram features, are mainly used to carry out some statistical calculations on the whole image or the ROI in the image, which are used to describe the image at the gray level. Second-order statistics involve the spatial relationship between each voxel intensity. Higher-order statistics are used for feature extraction and image preprocessing, such as wavelet decomposition, Fourier transform, and other filtering. Radiomics features were automatically extracted by the software, which made up for mistakes caused by manual and subjective measurement. In our results, the AUCs of radiomics nomogram, combined clinical and radiomics features, were 1.00 and 0.99 in training and validation sets, respectively. The AUCs of nomogram models were larger than that both clinical model and radiomics signature model, and there were statistically significant differences among the above three models. Feng et al. (17) developed a CT-based deep learning nomogram to identify tuberculous granuloma and LAC in patients with PSSN, the AUCs in training, internal validation and external validation sets were 0.889 (95% CI, 0.839-0.927), 0.879 (95% CI, 0.939-0.993) and 0.809 (95% CI, 0.746-0.862), respectively. Feng et al. (16) also developed another CT-based nomogram model using different patient numbers to identify LTB and LAC in patients with PSSN; the AUCs in training, internal validation, and external validation sets were 0.966 (95% CI, 0.839–0.927), 0.934 (95% CI, 0.894–0.974), and 0.906 (95% CI, 0.864–0.949), respectively. Another study found that radiomics nomogram had the potential to distinguish between LAC and granulomatous lesions; the AUCs in training and validation sets were 0.885 (95% CI, 0.823–0.931) and 0.808 (95% CI, 0.690–0.896), respectively (33). Compared to the results of the above studies, the prediction ability of our nomogram model was slightly better. All of these studies included clinical data in addition to the extraction of radiomics parameters in the model-building process, such as age, sex, nodule size, and other CT features. Before extracting parameters, we used 14 filters to process CT image, then we extracted 2,589 radiomics parameters for each ROI. This was different from the above three studies, which might provide different pieces of information for building prediction model. In addition, the patient population ratio of LAC and LTB was about 1:2 in our study, while the ratio of LAC and LTB patients was roughly 1:1 in other studies, which may mean that different data compositions could also influence the experimental results.

There were several limitations in this study. First, sample selection bias. Second, this was a two-center retrospective study, and the predictive ability was good, which may suggest that we need an external validation set to validate this predictive model. Third, this study involved two different CT machines, and the images had not been normalized, which may affect the study results. Fourth, we only evaluated the relationship between LAC and LTB, and other pathological types of lung nodules also needed further investigation, such as lung squamous cell carcinoma and other benign granulomatous lesions.

In conclusion, the radiomics nomogram, derived from unenhanced thin-slice chest CT images, showed favorable prediction efficacy for differentiating LAC from LTB in patients with lung solitary solid nodule.

## Data Availability Statement

The raw data supporting the conclusions of this article will be made available by the authors without undue reservation.

## Ethics Statement

The Ethic Review Boards of Shanghai Public Health Clinical Center and Zhongshan Hospital have approved this retrospective study and waived the written informed consent.

## Author Contributions

Guarantor of integrity of the entire study: FS. Study concepts and design: YaZ and YiZ. Literature research: YaZ. Clinical studies: JS and DW. Experimental studies/data analysis: MY. Statistical analysis: YaZ. <anuscript preparation: FS and ZZ. Manuscript editing: YaZ and YiZ. All authors contributed to the article and approved the submitted version.

## Funding

This work was supported by the Ningbo Medical Science and Technology Project (grant number 2018A14), the Intelligent Medical Special Research Foundation of the Shanghai Health and Family Planning Commission (grant number 2018ZHYL0104), and the Clinical Research Plan of SHDC (grant number SHDC2020CR3080B).

## Conflict of Interest

Author DW was employed by the company Shanghai United Imaging Intelligence Co., Ltd.

The remaining authors declare that the research was conducted in the absence of any commercial or financial relationships that could be construed as a potential conflict of interest.

The handling editor declared a shared affiliation with several of the authors YaZ, YiZ, ZZ, FS, JS at time of review.

## Publisher’s Note

All claims expressed in this article are solely those of the authors and do not necessarily represent those of their affiliated organizations, or those of the publisher, the editors and the reviewers. Any product that may be evaluated in this article, or claim that may be made by its manufacturer, is not guaranteed or endorsed by the publisher.
