# Supplementary material for: Clinical and CT Radiomics Nomogram for Preoperative Differentiation of Pulmonary Adenocarcinoma From Tuberculoma in Solitary Solid Nodule
Source: Front Oncol. 2021 Oct 12;11:701598. doi: 10.3389/fonc.2021.701598 (PMC8546326; doi:10.3389/fonc.2021.701598)
Supplement: Supplementary file 1 [file DataSheet_1.docx]

Supplementary materials

S1. Radscore calculation formula.

S2. Radiomics signature construction. Radscores were compared from lung adenocarcinoma (LAC) and lung tuberculoma (LTB) on training (A) and validation (B) set. ROC analysis was used to evaluate the performance of the model on training (C) and validation (D) set. 0: LAC; 1: LTB.

S3. Univariate logistic regression analysis of the clinical data.

| Variable units | Odds ratio | Lower | Upper | *P* value |
| --- | --- | --- | --- | --- |
| Spiculated | 2.933507e-01 | 0.13483455 | 0.63822397 | 1.986419e-03 |
| Lobulated | 1.176471e-02 | 0.00159166 | 0.08695847 | 1.342826e-05 |
| Vacuole | 2.027027e-01 | 0.05882670 | 0.69846488 | 1.145480e-02 |
| Boundary | 3.837108e+07 | 0.00000000 | Inf | 9.857774e-01 |
| Maxdiameter | 8.894001e-01 | 0.83676625 | 0.94534461 | 1.660064e-04 |
| Mindiameter | 8.312861e-01 | 0.76537067 | 0.90287827 | 1.166104e-05 |
| Meandiameter | 8.538667e-01 | 0.79338307 | 0.91896125 | 2.503190e-05 |
| Airbronchogram | 5.749386e-01 | 0.31858877 | 1.03755812 | 6.612757e-02 |
| Vessel | 3.674309e-01 | 0.13501669 | 0.99991701 | 4.998101e-02 |
| Lymphadenovarix | 2.759197e+00 | 1.38896296 | 5.48118998 | 3.753601e-03 |
| Age | 9.474124e-01 | 0.92903652 | 0.96615171 | 6.454467e-08 |
| Sex | 2.521886e+00 | 1.39874336 | 4.54687170 | 2.099347e-03 |

S4. Multivariate logistic regression analysis of the clinical data, to select the independent predictors of differential diagnosis between LAC and LTB.

| Variable units | Odds ratio | 95% CI | *P* value |
| --- | --- | --- | --- |
| Lobulated | 0.01 | [0.00;0.11] | < 1e-04 |
| Vacuole | 0.14 | [0.02;0.77] | 0.024054 |
| Mindiameter | 0.88 | [0.79;0.98] | 0.020139 |
| Lymphadenovarix | 2.66 | [1.03;6.85] | 0.043208 |
| Age | 0.95 | [0.93;0.97] | < 1e-04 |
| Sex | 4.13 | [1.74;9.77] | 0.001268 |

Note: LAC: lung adenocarcinoma; LTB: lung tuberculoma; CI: confidence interval.
