# Supplementary figures and images for: Clinical and CT Radiomics Nomogram for Preoperative Differentiation of Pulmonary Adenocarcinoma From Tuberculoma in Solitary Solid Nodule
Source: Front Oncol. 2021 Oct 12;11:701598. doi: 10.3389/fonc.2021.701598 (PMC8546326; doi:10.3389/fonc.2021.701598)

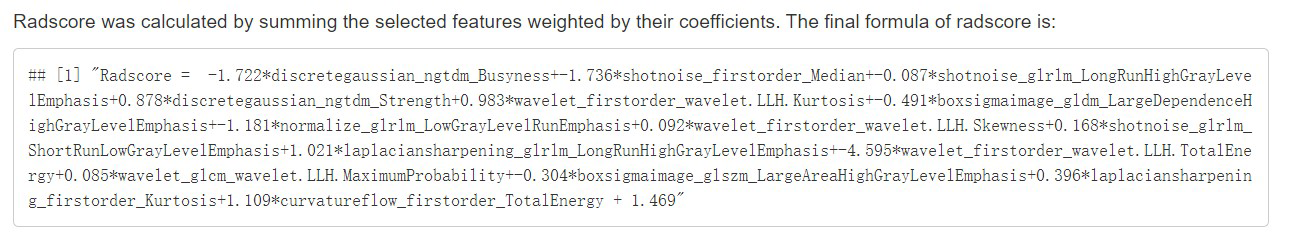

Supplement: Supplementary file 2 [file Image_1.tif]

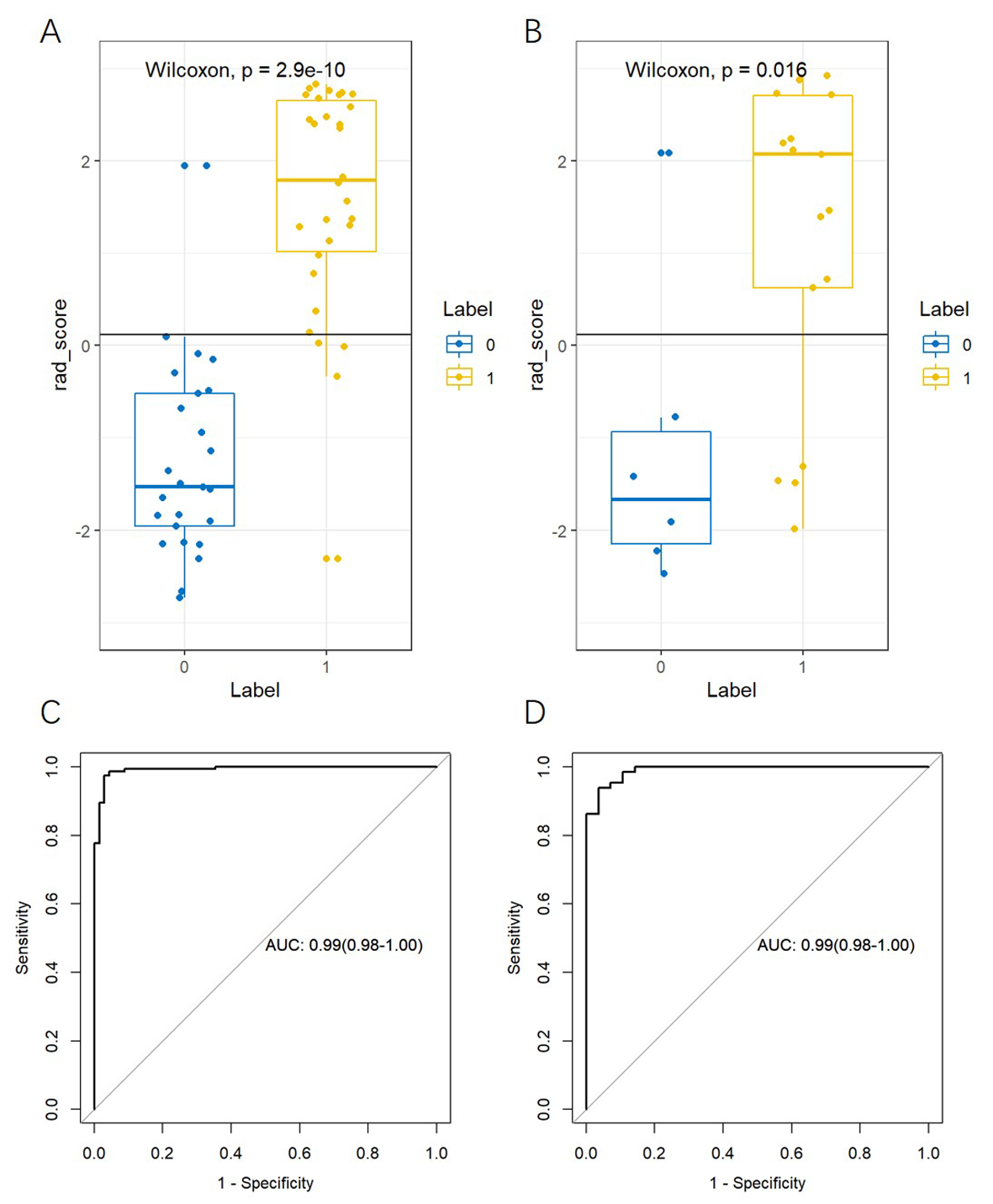

Supplement: Supplementary file 3 [file Image_2.tif]
